# Dental Diseases in Animals

**Published:** 1885-06

**Authors:** 


### DENTAL DISEASES IN ANIMALS.

At the meeting of the Odontological Society of Great Britain, held in London, April 13, 1885, (see Transactions, April No.) a very interesting paper was read by J. Bland Sutton, F. R. C. S., upon "Injuries and Diseases of the Jaws of Animals," in which he demonstrated, by means of actual specimens, that many animals are not exempt from dental and oral lesions. A number of cases of malformations, atrophies, hypertrophies, and morbid growths were exhibited. It was stated that some wild animals were quite liable to dental troubles of all kinds, such as caries, deposits of tartar, inflammation of the pulp, alveolar abscess, hypertrophies and necrosis, and these assertions were verified by the exhibition of confirmatory specimens. The skull of a monkey was produced which, it was said, had died from pyæmia induced by pyorrhea alveolaris. Odontomes and dentigerous cysts were shown as having occurred in both wild and domestic animals.

Mr. Sutton is rapidly gaining an enviable reputation as a comparative pathologist, and we shall look with interest for further contributions from him to this department of medical and dental science.

